# A novel high sensitivity UPLC-MS/MS method for the evaluation of bisphenol A leaching from dental materials

**DOI:** 10.1038/s41598-018-24815-z

**Published:** 2018-05-03

**Authors:** Siemon De Nys, Eveline Putzeys, Philippe Vervliet, Adrian Covaci, Imke Boonen, Marc Elskens, Jeroen Vanoirbeek, Lode Godderis, Bart Van Meerbeek, Kirsten L. Van Landuyt, Radu Corneliu Duca

**Affiliations:** 1 KU Leuven (University of Leuven), Department of Oral Health Sciences, BIOMAT & University Hospitals Leuven (UZ Leuven), Dentistry, Leuven, Belgium; 2 Toxicological Centre, University of Antwerp, Universiteitsplein 1, D.S.551, 2610 Wilrijk, Belgium; 3 Department of Analytical, Environmental and Geo-Chemistry, Vrije Universiteit Brussel, Pleinlaan 2, 1050 Ixelles, Belgium; 4 Environment and Health, Department of Public Health and Primary Care, KU Leuven, Kapucijnenvoer 35, 3000 Leuven, Belgium; 5 IDEWE, External service for prevention and protection at work, Heverlee, Belgium

## Abstract

There is a growing necessity to acquire more profound knowledge on the quantity of eluates from resin-based dental materials, especially with regard to bisphenol A (BPA). The aim of the present study was to develop a highly sensitive method to characterize the short-term release of BPA in saliva with ultra-performance liquid chromatography-tandem mass spectrometry (UPLC-MS/MS), using an extraction step and additional derivatization of BPA with pyridine-3-sulfonyl chloride. Light-cured resin-based composites were incubated at 37 °C in 1 mL artificial saliva, which was refreshed daily for one week. The final protocol allows accurate quantification of very low levels of BPA in samples of artificial saliva (i.e. 1.10 pmol BPA/mL or 250 pg/mL). The daily BPA-release from dental composites, ranging from 1.10 to 7.46 pmol BPA/mL, was characterized over a period of 7 days. The highest total amount of BPA was released from Solitaire 2 (24.72 ± 2.86 pmol), followed by G-ænial Posterior (15.51 ± 0.88 pmol) and Filtek Supreme XTE (12.00 ± 1.31 pmol). In contrast, only trace amounts of BPA were released from Ceram.x Universal. This UPLC-MS/MS method might be used for clinical research focusing on the evaluation of the clinical relevance of BPA release from dental materials.

## Introduction

A new field of interest in dentistry, namely endocrine active substances (EAS), has emerged since it was shown for the first time ever that bisphenol A (BPA) and BPA-derivatives, detected in saliva from patients treated with resin-based dental sealants, were causing estrogenic activity *in vitro*^1^. EAS are chemicals that interact and/or interfere with the endocrine system through altering physiological hormone levels and mimicking or blocking endogenous hormones at all possible biological levels, from synthesis to effects in target tissue. When adverse reactions such as developmental, reproductive, neurological, cardiovascular, immune and metabolic effects occur, these EAS are called endocrine disrupting chemicals (EDCs) or endocrine disruptors^2,3^. The estrogenic activity of BPA is already well known for a long time and is linked to several diseases including reproductive, developmental, metabolic problems and even hormonal cancers^3^.

BPA is used for the synthesis of several methacrylate monomers, such as (urethane-modified) BisGMA, BisEMA, BisDMA, BisPMA, and BADGE. Therefore, BPA is most likely present as an impurity of the production process of these monomers, which can potentially be released into the oral cavity after light-curing, followed by ingestion. Furthermore, biodegradation and mechanical degradation of the dental material and monomers may increase this release^4^. Monomers with a BPA-core are commonly used as major monomer in resin-based materials such as sealants, adhesives, composites and root canal sealers that are used for preventive, (temporary) restorative, orthodontic or endodontic applications. From all leached and detected ingredients from resin-based materials, BPA has led to the most controversy due to its endocrine disrupting nature.

The *in-vitro* release and associated estrogenic effects of BPA has already been reviewed extensively^4–8^. However, there is still no conclusive evidence about the relevance of *in-vivo* BPA exposure due to BPA released from dental materials compared to other sources, such as diet (food and beverage containers), dust and thermal paper^9^, and whether the detected levels are sufficient to induce estrogenic effects *in vivo*. There is evidence that BPA, and EDCs in general, may exert non-monotonic dose-responses^10–12^, which indicates that even low doses (nanomolar range) of BPA may induce adverse effects although these levels are assumed to be safe. Moreover, the activity level lies within a range that is under the detection limit of most analytical methods^13^.

Several studies have shown that BPA can be released on short term *in vivo*^14–17^. Increased salivary BPA levels (picomolar range) have been observed within one hour after placement of a dental restoration, but steadily decreased back to baseline levels^14,15^. However, there is still no conclusive evidence whether BPA is released *in vivo* in the long term. Moreover, due to the endocrine disrupting activity, which is even observed at very low concentrations, the debate is still ongoing whether dental materials should be regarded as a relevant source of BPA.

Many analytical methods have proven their value in elucidating this topic. However, comparing between studies is usual difficult due to various differences in study design. There is no standardized analytical method for BPA and crucial information about the applied method is seldom provided. Earlier studies assessing *in-vivo* BPA release after placement of sealants and/or composites used HPLC, often in combination with a UV-VIS (ultraviolet-visible light) detector^1,17–19^. However, this technique has severe limitations regarding the specificity and sensitivity. UV detection is based on the absorbance of a beam of light with a certain wavelength. This method cannot distinguish molecules based on their molecular weight and thus interferences are possible if proper separation of molecules is not achieved. It was shown that BPA co-eluted with another compound that was most likely derived from one of the photo-initiators, thereby overestimating the actual amount of released BPA as detected by UV^20^. Another study showed that BisGMA showed a similar retention time as BPA^21^. This problem can be solved by ultraperformance liquid chromatography-tandem mass spectrometry (UPLC-MS/MS). This technique is based on the detection of the mass over charge ratio of a compound of interest and its daughter ions, leading to two extra parameters that are compound-specific. Furthermore, the use of a deuterated internal standard (IS) allows comparison of retention times as one additional control, which have to be different for UV detection. Other studies assessed BPA release in saliva using ELISA^15,22,23^. However, also this technique was not very reliable in biological samples due to various reasons such as cross-reactivity of the anti-BPA antibodies^24^. Techniques such as HPLC and GC-MS are relatively slow, with run times up to several tens of minutes.

Nevertheless, there is an increasing need towards more sensitive and specific methods necessary for an accurate quantification of lower levels of BPA, down to picomolars, to elucidate the concerns about the exposure of BPA from dental materials, especially with regard to children, who are more vulnerable to these endocrine effects. Therefore, this study describes the development of an accurate and sensitive UPLC-MS/MS detection method for low levels of BPA, based on a sample preparation step and additional derivatization of BPA with pyridine-3-sulfonyl (PS) chloride. The optimized highly sensitive method was applied to a one-week elution experiment using resin-based dental composites and might also further be applied for a quick and accurate quantification of BPA released from dental materials in saliva *in vivo*.

## Results

### Method validation

#### Linearity and limits of quantification

The calibration curve of derivatized BPA with stable isotope labelled IS was linear within the domain of calibration (1.10–43.80 pmol BPA/mL) (Table 1). The correlation coefficient R^2^ of the regression equations exceeded the value 0.99. Appropriate values for both accuracy and precision were obtained for all calibration concentrations ranging between LLOQ and HLOQ. These results indicated a good correlation between the measured response (peak area) and the nominal concentration of BPA. The limit of detection (LOD) was 0.44 pmol BPA/mL.

**Table 1:** Intra-assay and inter-assay validation results.

Compound	Linearity (R^2^)	*LLOQ-HLOQ* (pmol/mL)	Intra-assay (n = 5)						Inter-assay (n = 3)					
			Accuracy (% of target)			Precision (RSD%)			Accuracy (% of target)			Precision (RSD%)		
			*Level 1* ^a^	*Level 2* ^*b*^	*Level 3* ^*c*^	*Level 1* ^a^	*Level 2* ^*b*^	*Level 3* ^*c*^	*Level 1* ^a^	*Level 2* ^*b*^	*Level 3* ^*c*^	*Level 1* ^a^	*Level 2* ^*b*^	*Level 3* ^*c*^
BPA	0.9976	1.10–43.80	96	101	99	15	4	4	92	101	100	10	3	3

^a^Level 1: 1.10 pmol BPA/mL, ^b^Level 2: 11.00 pmol BPA/mL, ^c^Level 3: 43.80 pmol BPA/mL.

Abbreviations: LLOQ: lower limit of quantification; HLOQ: higher limit of quantification; RSD: relative standard deviation.

#### Intra- and inter-assay accuracy and precision

Accuracy and precision were evaluated at three different concentrations covering the range of calibration. Intra-assay accuracy varied between 96% and 101% of the target. Intra-assay precision ranged between 4% and 15% RSD. The inter-assay accuracy varied between 92% and 101%, while the inter-assay precision ranged between 3% and 10% RSD (Table 1). These results indicated that the method is highly reproducible and accurate.

### BPA leaching experiment

**Figure 1:**
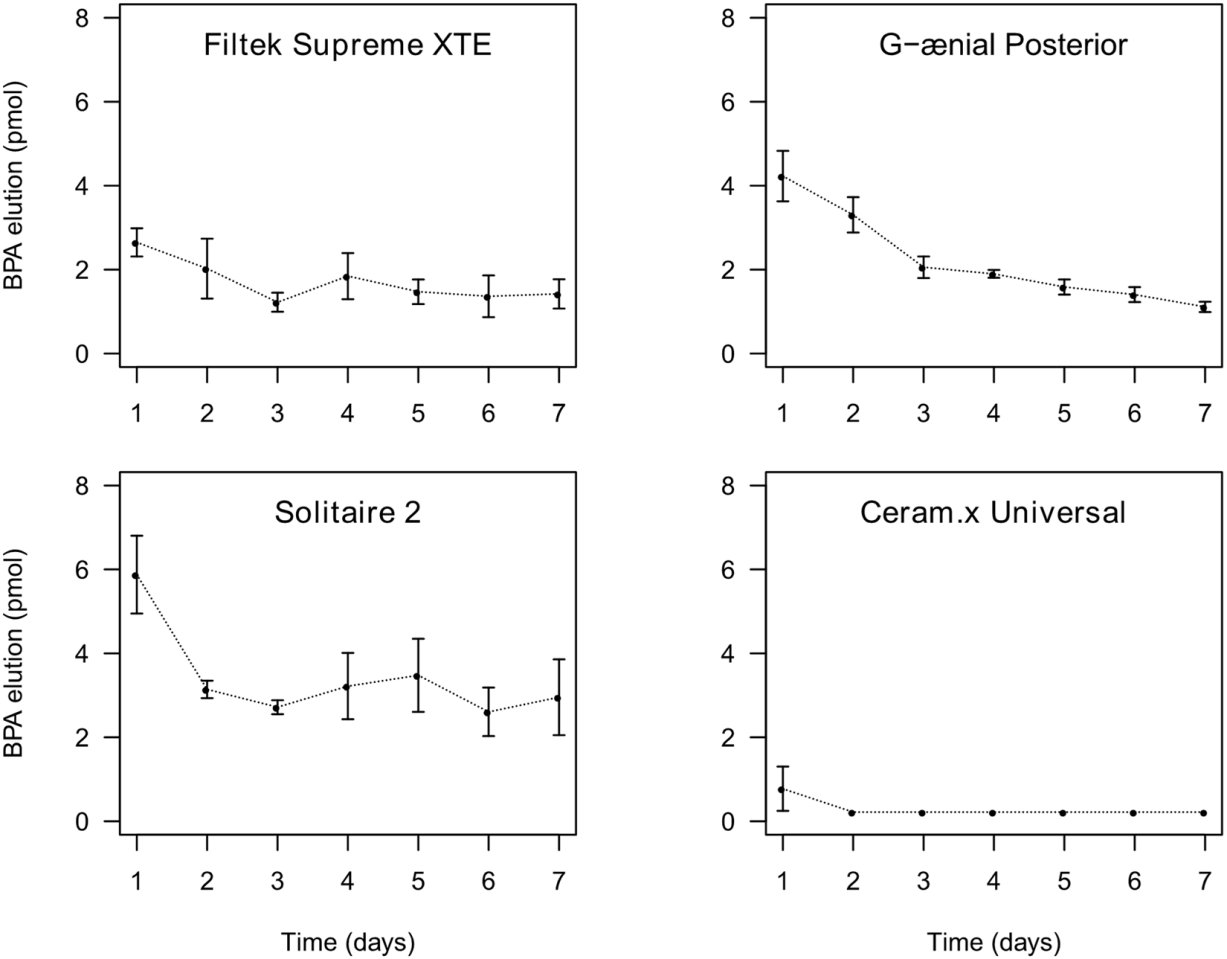
Released quantities of BPA from resin-based dental materials over a period of 7 days, expressed in mean ± SD (n = 5). Calculated values under LOD are substituted by LOD/2.

BPA was released in detectable amounts from all materials, except Ceram.x Universal, during the whole study period (Fig. 1; Table 2). Solitaire 2 released the highest cumulative amount of BPA (0.424 ± 0.046 pmol BPA/mm²), ranging from 0.101 ± 0.016 pmol BPA/mm² after one day to 0.051 ± 0.015 pmol BPA/mm² after one week, followed by G-ænial Posterior (0.266 ± 0.015 pmol BPA/mm²), ranging from 0.073 ± 0.010 pmol BPA/mm² after one day to 0.019 ± 0.002 pmol BPA/mm² after one week. Filtek Supreme XTE released 0.045 ± 0.066 pmol BPA/mm² after one day and still 0.024 ± 0.006 pmol BPA/mm² after one week, resulting in a cumulative release of 0.206 ± 0.023 pmol BPA/mm². Trace amounts of BPA (0.014 ± 0.009 pmol BPA/mm²) were detected in samples of Ceram.x Universal after one day only, even though these levels were below the reported LLOQ, but above the LOD, leading to an inaccurate quantification.

**Table 2:** Released BPA quantities eluted from resin-based dental materials over a period of 7 days.

Composite	Release of BPA (pmol/mm²)							
	Day 1	Day 2	Day 3	Day 4	Day 5	Day 6	Day 7	*Total*
Filtek Supreme XTE	0.045 ± 0.006	0.035 ± 0.012	0.021 ± 0.004	0.032 ± 0.009	0.025 ± 0.005	0.023 ± 0.009	0.024 ± 0.006	*0*.*206 ± 0*.*023*
G-ænial Posterior	0.073 ± 0.010	0.057 ± 0.007	0.035 ± 0.004	0.033 ± 0.002	0.027 ± 0.003	0.024 ± 0.003	0.019 ± 0.002	*0*.*266 ± 0*.*015*
Solitaire 2	0.101 ± 0.016	0.054 ± 0.004	0.047 ± 0.003	0.055 ± 0.014	0.060 ± 0.015	0.045 ± 0.010	0.051 ± 0.015	*0*.*424 ± 0*.*046*
Ceram.x Universal	<LLOQ	<LLOQ	<LLOQ	<LLOQ	<LLOQ	<LLOQ	<LLOQ	*/*

Values are mean ± SD, n = 5. (<LLOQ: below lower limit of quantification (i.e. 0.03 pmol BPA/mm²); /: no accurate quantification possible).

## Discussion

UPLC-MS/MS has become one of the most widely used and valuable detection methods in the analysis of compounds present in trace amounts due to its sensitivity and selectivity, compared to other analytical techniques, such as HPLC(-UV/VIS) and GC-MS. However, the physico-chemical properties of BPA limit its ionization, which is essential for MS detection. Chemical derivatization can overcome this limitation by adapting the physico-chemical properties of BPA, thereby increasing the ionization efficiency allowing more BPA molecules to be detected compared to non-derivatized BPA^25^. Furthermore, by increasing the molecular mass of the analytes, the signal-to-noise ratio is also increased. Both features result in a higher sensitivity^26^. To our knowledge, derivatization has rarely been applied in the field of dental research^27^. One study used BSTFA + TMCS for derivatization of BPA released from orthodontic retainers in combination with GC-MS^28^. This procedure, using PS chloride for BPA derivatization, allowed us to further reduce the LLOQ (i.e. 1.10 pmol BPA/mL), which is 200 times lower than the previously published method by our research group (i.e. 219.02 pmol BPA/mL)^29^. However, it should be noted that this latter method was developed not only for analysis of BPA, but also for as much as 12 other (BPA-based) monomers and other common ingredients of dental materials. In addition, our method can analyse one sample within 2.5 minutes, which allows high throughput and makes this method very suitable for large epidemiological studies.

BPA was released in detectable amounts from three out of four tested materials over a 7-day period after incubation of the composite samples in artificial saliva (AS) to simulate a clinically relevant situation. As expected, higher amounts were detected in the first few days, followed by a consistent lower release in the next days (Fig. 1; Table 2). The differences between the composites can be explained by the amount of BPA-based monomers in the resin and their physico-chemical properties (solubility in the water-based AS). Furthermore, other parameters such as the degree of conversion are also known to play a role in the release of monomers, although this was not tested in this study^30^.

No BPA, in levels above the LLOQ after subtracting the procedural blanks, was detected in extracts from Ceram.x Universal, which hindered an accurate quantification. One possible explanation is the recent development of new filler technology that binds more free resin compared to standard fillers, which could hinder the release of monomers and BPA. Hence, the presence of a BPA-based monomer in the raw material does not necessarily indicate that BPA will be released in detectable amounts from this certain material after light-curing. Therefore, every material should be evaluated independently. Manufacturers should also focus on the production of BPA-free materials to avoid the presence of background contamination in order to accurately quantify even lower levels of BPA. In addition, this might point out the need for even more sensitive detection methods.

Based on these results, the approximately average daily release from resin-based composites was 0.043 pmol BPA/mm². Specimens had a total exposed surface of 58.3 mm² (top and side surfaces), which corresponds to the total exposed surface area of one mesial-occlusal-distal box restoration (i.e. on average 60 mm²)^4^. Moreover, wear-patients (i.e. tooth loss due to attrition, abrasion and erosion) typically require full crown restorations of all teeth. In this worst-case scenario, the total exposed surface area of is 7372 mm², which would yield to a daily release of 314.44 pmol BPA. This may suggest that, especially in patients with multiple large restorations, resin-based dental composites may present a relevant source of BPA exposure compared to other sources such as food and beverage cans (0.4–4.2 µg/kg bw/day for adults), dust (less than 0.006 µg/kg bw/day and 0.0005 µg/kg bw/day for toddlers and adults, respectively) and thermal paper (worst case of 0.97 µg/kg bw/day)^31–33^. Nevertheless, whether BPA is released *in vivo* over longer periods remains unknown and can only be assessed in large epidemiological studies in which this procedure might be applied for analysing BPA release in saliva and urine samples. In addition, care should be taken since one cannot distinguish between BPA proportions coming from the different sources due to its widespread use.

## Conclusions

We describe in this study a highly sensitive and accurate method to detect low levels of BPA, released from resin-based dental materials, in artificial saliva. To our knowledge, this is the first time a derivatization reagent was used in combination with UPLC-MS/MS for the analysis of BPA released from dental materials. Using derivatization with PS chloride allowed us to further reduce the limit of detection and quantification (i.e. 0.44 and 1.10 pmol BPA/mL, respectively) and accurately quantify the release of BPA, which makes this method suitable for biomonitoring studies aiming at BPA quantification in biological samples such as saliva. These results indicate that resin-based composites may be considered as sources of BPA, which might be relevant in patients with multiple large restorations.

## Materials and Methods

### Chemicals and materials

BPA (≥99%), the internal standard (IS) deuterated BPA-d_16_ (analytical standard), the derivatization reagent pyridine-3-sulfonyl (PS) chloride (≥95%), acetonitrile (ACN, LC-MS grade), dichloromethane (DCM, LC-MS grade), methanol (MeOH, LC-MS grade), and formic acid (FA, LC-MS grade) were purchased from Sigma-Aldrich (Diegem, Belgium). H_2_O (LC-MS grade) was purchased from Biosolve (Valkenswaard, The Netherlands). Oasis PRiME HLB (3 mL; 60 mg) extraction cartridges were purchased from Waters (Zellik, Belgium).

Filtek Supreme XTE was obtained from 3M ESPE (Seefeld, Germany). G-ænial Posterior was obtained from GC Europe (Leuven, Belgium). Solitaire 2 was obtained from Kulzer (Hanau, Germany). Ceram.x Universal was obtained from Dentsply (Konstanz, Germany). An overview of these resin-based dental composites is given in Table 3. This selection was based on the presence of at least one BPA-based monomer in the resin.

**Table 3:** List of the composites investigated and their composition as provided by the manufacturer’s safety data sheets.

Composite	Composition		Manufacturer
*shade A3*	*Monomer (wt*.*%)*	*CAS*	
Filtek Supreme XTE	BisGMA* (1–10%) UDMA (1–10%) TEGDMA (<5%) BisEMA* (1–10%) PEGDMA (<5%)	1565–94–2 72869–86–4 109–16–0 41637–38–1 25852–47–5	3M ESPE, Seefeld, Germany
G-ænial Posterior	UDMA (10–25%) TCDDMA (2.5–5%) BisEMA* (1–2.5%)	72869–86–4 43048–08–4 41637–38–1	GC Europe, Leuven, Belgium
Solitaire 2	BADGE-DA* (5–10%) TEGDMA (5–10%)	55818–57–0 109–16–0	Kulzer, Hanau, Germany
Ceram.x Universal	BisEMA* (2.5–10%) TEGDMA (2.5–10%) urethane-modified BisGMA dimethacrylate resin* (2.5–10%)	41637–38–1 109-16-0 126646-17-1	Dentsply, Konstanz, Germany

^*^BPA-based monomer.

Abbreviations: BADGE-DA: bisphenol A diglycidyl ether diacrylate; BisEMA: ethoxylated bisphenol A glycol dimethacrylate; BisGMA: bisphenol A diglycidyl dimethacrylate; PEGDMA: polyethylene glycol dimethacrylate; TCDDMA: tricyclodecanedimethanol dimethacrylate; TEGDMA: triethylene glycol dimethacrylate; UDMA: urethane dimethacrylate.

### Apparatus and operating conditions

A Xevo TQ-XS Triple Quadrupole Mass Spectrometer (Waters, Zellik, Belgium) equipped with electrospray ionization (ESI) was used for all sample analyses. The source temperature was set at 150 °C and the desolvation temperature was set at 600 °C. Argon was used as the collision gas (0.15 mL/min) and nitrogen as the desolvation gas (1000 L/h). Nitrogen was also used as nebulization gas (7 bar). Multiple reaction monitoring (MRM) parameters for the analysis of the target compound BPA and the IS are summarized in Table 4. For each compound, the MRM transition with the highest measured response was set as the ‘quantifier transition’. Other transitions were used as ‘qualifier transitions’ for confirmation. Analysis was performed in positive ionization mode.

**Table 4:** MS/MS parameters for the analysis of derivatized BPA and derivatized IS. Abbreviations: R_t_: retention time; ES+: positive electrospray ionisation.

Compound	R_t_ (min)	Ionization mode	Quantifier			Qualifier		
			Transition	Cone (V)	Collision (V)	Transition	Cone (V)	Collision (V)
BPA-diPS	1.75	ES+	511 → 354	10	30	354 → 290	10	35
						354 → 212	10	44
BPA-d_14_-diPS (IS)	1.75	ES+	525 → 301	10	40	286 → 144	10	40

Standard mixtures and samples (10 µL) were injected into an Acquity UPLC BEH C18 column (50 mm × 2.1 mm, 1.7 µm; Waters), kept at a temperature of 40 °C. Chromatographic conditions were, using a mixture of H_2_O (0.1% FA, solvent A) and MeOH (0.1% FA, solvent B) as mobile phase, as follows: 0–0.2 min, 40% B; 0.2–0.7 min, 40–95% B; 0.7-1.7 min, 95% B; 1.7–2.1 min, 95–40% B; 2.1–2.5 min, 40% B. The flow rate was set at 0.400 mL/min.

### Method performance and validation

The developed UPLC-MS/MS method was validated for linearity of the calibration curves and the associated correlation coefficient R², lower limit of quantification (LLOQ), higher limit of quantification (HLOQ), and intra- and inter-assay accuracy and precision in accordance with international rules^34^.

#### Linearity and limits of quantification

Calibration standards containing 102.30 pmol BPA-d_16_ were freshly prepared and immediately analysed. The calibration curve was fitted by linear regression analysis on the peak area ratios of the target compound to the IS, against the nominal concentration of the target compounds. Fitting of the calibration curve was evaluated using the error on the calculated concentrations expressed as percentage of target concentrations (% of target) and the correlation coefficient R^2^ of the calibration curve. These calibration standards were processed exactly as the samples from the leaching experiment. Standard blanks were used to correct for background contamination.

#### Intra-assay and inter-assay accuracy and precision

To ensure correct quantification, intra-assay accuracy and precision were evaluated by determining the compound concentrations in five replicates at low, medium and high concentration levels with the lowest level being the LLOQ and the highest level being the HLOQ. Calibration standards were injected five times. The intra-assay accuracy was calculated as the error to the nominal concentrations (% of target). The intra-assay precision was calculated as the relative standard deviation (RSD%). Inter-assay accuracy and precision were assessed by analysing the calibration standards on three separate days. The precision determined at each concentration should not exceed 20% while the accuracy should be within 80–120%.

#### Selectivity

Derivatized BPA was analysed using compound-specific MRM, leading to a reduction of interferences from other compounds in the sample. Moreover, besides a quantification transition (‘quantifier’), at least one additional transition (‘qualifier’) was used for the confirmation of the target compound. Additionally, correct identification was ensured by checking the target specific retention time (Table 4).

### Preparation of artificial saliva

Artificial saliva (AS) was prepared as described previously^35^. Briefly, 1 L artificial saliva contains 896 mg potassium chloride (KCl), 888 mg sodium phosphate monobasic dehydrate (NaH_2_PO_4_), 200 mg potassium thiocyanate (KSCN), 298 mg sodium chloride (NaCl), 1.8 mL sodium hydroxide (NaOH) (1 M), 200 mg urea, 15 mg uric acid, 50 mg mucin and 145 mg α-amylase.

### BPA leaching experiment

Specimen disks (5.5 mm diameter and 2 mm thickness; surface area 58.3 mm²) were prepared in a custom-made Teflon mould. The top and bottom were covered by a glass plate to prevent oxygen inhibition, to ensure smooth surfaces and to avoid excess of material. Samples were polymerized for 20 s by light-curing using a LED light-curing unit (Demi Ultra, Kerr, CA, USA) with minimal light intensity of 1700 mW/cm², as measured by the MARC resin calibrator (BlueLight Analytics, Halifax, NS, Canada). Subsequently, the disks (n = 5 for each composite type) were immediately immersed in 1 mL AS containing isotope-labelled BPA-d_16_ as IS (102.30 pmol). Samples were incubated for 1 week at 37 °C. The extraction solvent was renewed daily to simulate the continuous flow of saliva. Next, samples were extracted followed by derivatization. To avoid contamination, care was taken to use only glass pipettes and glass containers whenever possible. Additionally, procedural blanks were used to correct for background contamination.

### Solid phase extraction (SPE)

In order to purify BPA and remove as much as possible impurities from the AS that could interfere with MS analysis, solid phase extraction was performed as previously by Geens *et al*., with minor modifications^36^. An Oasis PRiME HLB cartridge (3 cc, 60 mg) was prewashed with 1 × 3 mL DCM and 1 × 3 mL MeOH, and subsequently conditioned with first 1 mL H_2_O, and again with 2 mL. Samples were acidified with 1% FA (1:1) and subsequently loaded into the cartridge and washed with 2 mL H_2_O. Next, BPA was eluted with 2 × 2.5 mL DCM/MeOH (1:1) under vacuum (10 mmHg).

### Derivatization procedure

Following sample extraction, BPA was derivatized using PS chloride prior to analysis as previously described^26^. Briefly, the SPE eluate was evaporated to dryness under a gentle nitrogen flow and reconstituted in 200 µL sodium carbonate buffer (50 mM, pH 9.8). Next, 200 µL derivatization reagent (1 mg/mL in ACN) was added. After incubation at 70 °C for 15 min, the reaction was stopped by cooling down the samples on ice and 100 µL FA (1 M) was added to obtain a final volume of 500 µL. Samples were filtered (0.20 µm regenerated cellulose filter) and stored at −20 °C until analysis.

### Data availability

All data generated or analysed during this study are included in this published article.
